# Molybdenum metabolism in plants and crosstalk to iron

**DOI:** 10.3389/fpls.2014.00028

**Published:** 2014-02-07

**Authors:** Florian Bittner

**Affiliations:** Department of Plant Biology, Braunschweig University of TechnologyBraunschweig, Germany

**Keywords:** aldehyde oxidase, iron, molybdenum, molybdate transporter, molybdo-enzymes, nitrate reductase, sulfite oxidase, xanthine dehydrogenase

## Abstract

In the form of molybdate the transition metal molybdenum is essential for plants as it is required by a number of enzymes that catalyze key reactions in nitrogen assimilation, purine degradation, phytohormone synthesis, and sulfite detoxification. However, molybdate itself is biologically inactive and needs to be complexed by a specific organic pterin in order to serve as a permanently bound prosthetic group, the molybdenum cofactor, for the socalled molybdo-enyzmes. While the synthesis of molybdenum cofactor has been intensively studied, only little is known about the uptake of molybdate by the roots, its transport to the shoot and its allocation and storage within the cell. Yet, recent evidence indicates that intracellular molybdate levels are tightly controlled by molybdate transporters, in particular during plant development. Moreover, a tight connection between molybdenum and iron metabolisms is presumed because (i) uptake mechanisms for molybdate and iron affect each other, (ii) most molybdo-enzymes do also require iron-containing redox groups such as iron-sulfur clusters or heme, (iii) molybdenum metabolism has recruited mechanisms typical for iron-sulfur cluster synthesis, and (iv) both molybdenum cofactor synthesis and extramitochondrial iron-sulfur proteins involve the function of a specific mitochondrial ABC-type transporter.

## INTRODUCTION

Molybdenum is a transition metal, which occurs in the lithosphere at an average abundance of 1.2 mg kg^-1^ and represents one of the scarcest trace elements in biological systems (Kaiser et al., 2005). In the soil, molybdenum exists predominantly in the form of the oxyanion molybdate, which serves as an essential micronutrient in all kingdoms of life. Yet, molybdate alone does not exhibit biological activity, but is bound to an organic pterin backbone, which upon binding of molybdate is converted into the molybdenum cofactor (Moco). Once being incorporated as prosthetic group, Moco becomes part of the active site of molybdo-enzymes, where molybdenum can vary its oxidation state between Mo(IV), Mo(V), and Mo(VI), thereby enabling the respective protein to transfer electrons, and in most cases also oxygen, from or to a substrate (Hille, 2013). Due to its special importance for plants, another molybdenum-containing cofactor exclusively found in certain bacteria is mentioned. This cofactor is part of the unique enzyme nitrogenase that catalyzes the fixation of nitrogen by reduction of atmospheric N_2_ to NH_3_ in free-living, but also symbiotic bacteria in the nodules of legumes (Kneip et al., 2007). Unlike Moco however, the nitrogenase cofactor is constituted of molybdenum ligated to a complex iron-sulfur cluster and homocitrate and therefore is named FeMoco (Hu and Ribbe, 2013).

In soil, a critical point concerns the bioavailability of molybdate, which is favored above pH 5.5 and impaired at lower pH due to the adsorption of molybdate to soil oxides (Kaiser et al., 2005). Under low-pH conditions, molybdate assimilation is therefore limited resulting in molybdenum deficiency associated with reduced molybdo-enzyme activities and reductions in plant growth and yield. Fortunately, this type of molybdenum deficiency can be compensated by fertilization with molybdate or by increasing the soil pH by liming. In contrast, molybdenum toxicity by oversupply of plants with molybdate is extremely rare and characterized by relatively mild symptoms such as yellowish leaves (Kaiser et al., 2005) or reduced seedling growth and increased anthocyanin concentrations (Kumchai et al., 2013).

**FIGURE 1:**
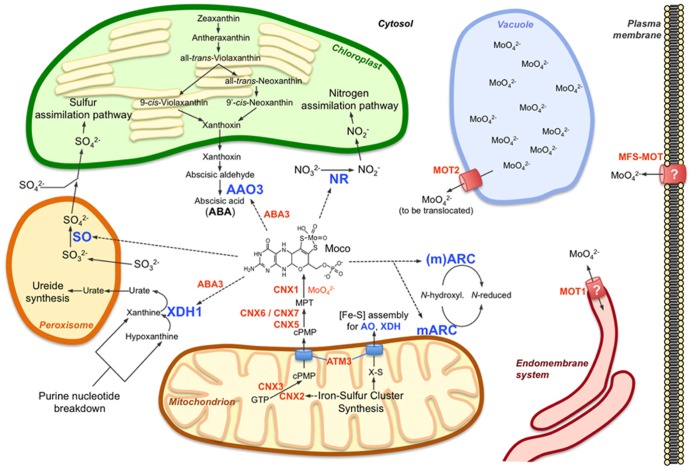
**Molybdenum metabolism in higher plant cells**. The main components of molybdenum metabolism in plants are shown including the Moco biosynthetic pathway (CNX proteins) in mitochondria and cytosol, the Moco user enzymes and their respective main functions in nitrogen assimilation (NR), ABA synthesis (AAO3), purine catabolism (XDH1), and sulfite detoxification (SO). mARC enzymes are proposed to function in reduction of certain N-hydroxylated substrates, which have not yet been identified. While one of the two mARC isoforms (mARC2) contains an NH_2_-terminal mitochondrial targeting sequence, such targeting sequence is absent at the second isoform, which therefore is assumed to act in the cytosol. In contrast to the molybdate transporter MOT2, which functions at the vacuolar membrane as a molybdate exporter, MOT1 might localize to the endomembrane system, possibly to the endoplasmic reticulum. A mitochondrial localization of MOT1 has also been reported but appears less likely as no obvious reason exists for import or export of molybdate into or out of the mitochondria, respectively. The plant homolog (MFS-MOT) of the major facilitator superfamily molybdate transporter MOT2 from Chlamydomonas might be required for molybdate import across the plasma membrane. The function of the Moco sulfurase ABA3 in activation of AO and XDH is indicated, just as the functions of the mitochondrial ABC transporter ATM3 in export of cPMP from mitochondria and in cytosolic iron-sulfur cluster ([Fe-S]) assembly for AO and XDH (and other extra-mitochondrial proteins). Further details are given in the main text and are reviewed by Bittner and Mendel (2010) and Mendel (2013). Molybdo-enzymes are indicated by blue letters, other components of molybdenum metabolism by orange letters; dotted arrows indicate requirement for Moco by molybdo-enzymes.

In consideration of the present knowledge of molybdate uptake, transport and storage, the specific functions of the molybdenum-dependent enzymes and the interrelation between molybdenum and iron, this review focuses on the current understanding of the molybdenum homeostasis network in plants and points to some hitherto unidentified factors (**Table 1**; **Figure 1**).

**Table 1:** Components of molybdenum metabolism in higher plants (*Arabidopsis thaliana*).

Protein names	Agi code	Known / proposed function
MOT1/SULTR 5;2	AT2G25680	Molybdate transport
MOT2/SULTR 5;1	AT1G80310	Molybdate transport / export from vacuole
CNX1	AT5G20990	Moco biosynthesis step 3
CNX2	AT2G31955	Moco biosynthesis step 1
CNX3	AT1G01290	Moco biosynthesis step 1
CNX5	AT5G55130	Moco biosynthesis step 2
CNX6	AT2G43760	Moco biosynthesis step 2
CNX7	AT4G10100	Moco biosynthesis step 2
Nia1/NR1	AT1G77760	Nitrate reductase (minor form)
Nia2/NR2	AT1G37130	Nitrate reductase (main form)
SO	AT3G01910	Oxidation/elimination of cytotoxic sulfite
mARC1/MOSC1	AT5G44720	Unknown (reduction of *N*-hydroxylated compounds ?)
mARC2/MOSC2	AT1G30910	Unknown (reduction of *N*-hydroxylated compounds ?)
AAO1	AT5G20960	Unknown (IAA biosynthesis ?)
AAO2	AT3G43600	Unknown (IAA biosynthesis ?)
AAO3	AT2G27150	ABA biosynthesis
AAO4	AT1G04580	Synthesis of benzoic acid
AtXDH1	AT4G34890	Purine degradation
AtXDH2	AT4G34900	Pseudogene ?
ABA3/LOS5	AT1G16540	Mocosulfuration and activation of AO and XDH proteins
ATM3/ABCB25	AT5G58270	Transporter involved in cytosolic Fe-S assembly and Moco synthesis

## UPTAKE, TRANSPORT, AND STORAGE OF MOLYBDATE

Early in plant molybdenum research Kannan and Ramani (1978) described the active uptake of exogenously applied molybdate by roots and its transport to the shoot. However, molybdate levels reached a maximum in the shoot already 6 h after application, indicating that uptake of molybdate and sensing of intracellular molybdate levels are well controlled processes. Notably, when molybdate is applied solely to leaves transport also occurs downward to the stem and roots, demonstrating that molybdate is a highly mobile compound translocated between various plant tissues. Furthermore, the finding that sulfate is a potent inhibitor of molybdate uptake (Stout et al., 1951; Kannan and Ramani, 1978) and that in turn low sulfate concentrations in the soil promote molybdate uptake (Shinmachi et al., 2010) pioneered speculations on the nature of molybdate transporters. In fact, sulfate and molybdate share a high degree of similarity as they both possess a double negative charge (SO_4_^2-^, MoO_4_^2-^), are similar in size, and have tetrahedral structures. It was thus proposed that molybdate import and distribution are facilitated by sulfate transporters or related systems. The first identified molybdate-specific transporters from *Chlamydomonas reinhardtii* (Tejada-Jimenez et al., 2007) and *Arabidopsis thaliana* (Tomatsu et al., 2007; Baxter et al., 2008) indeed turned out to belong to the family of sulfate transporters, yet lacking the otherwise typical STAS domain essential for sulfate transport activity (Shibagaki and Grossman, 2006). Unfortunately, the two reports on the *Arabidopsis* transporter, referred to as MOT1, differ in the subcellular localization of the transporter with Tomatsu et al. (2007) primarily showing endomembrane localization and Baxter et al. (2008) reporting a mitochondrial localization. Ascribing a precise function to MOT1 is therefore difficult and it remains to be proven in future studies whether MOT1 is involved in the intra- and/or the inter-cellular translocation of molybdate. In addition to MOT1, another molydate transporter of the sulfate transporter family, MOT2, has been identified in *Arabidopsis* (Gasber et al., 2011). MOT2 localizes to the vacuole and *MOT2*-deficient plants are characterized by accumulation of molybdenum in leaves and reduced molybdenum contents in seeds. Along with the finding that *MOT2* transcripts accumulate in senescing leaves, this suggests a function of MOT2 in exporting stored molybdate from the vacuole into the cytosol for translocation into maturing seeds. Moreover, total molybdenum contents were found to correlate with the levels of Moco in wildtype and *MOT2* mutant leaves, indicating plants adjust Moco synthesis to the cellular levels of molybdate.

Just recently a novel molybdate transporter, also denoted as MOT2, has been identified in *Chlamydomonas* (Tejada-Jiménez et al., 2011). In contrast to *Arabidopsis* MOT2 however, this transporter is a member of the major facilitator superfamily which is completely unrelated to the sulfate transporter family. It is tempting to speculate that the homolog of this transporter in higher plants represents the as yet unidentified transporter involved in the uptake of molybdate at the root:soil interface.

## MOLYBDENUM COFACTOR AND MOLYBDENUM-DEPENDENT ENZYMES

The only process known to require molybdenum/molybdate directly by plants is the biosynthesis of Moco, which is initiated in mitochondria and finalized in the cytosol. However, a detailed description of Moco biosynthesis is out of the scope of this work and is comprehensively covered by Bittner and Mendel (2010) and Mendel (2013). Even though Moco biosynthesis is well understood only little knowledge exists about the genetic and enzymatic regulation of Moco biosynthesis in plants. For instance, it is largely unknown whether the expression levels of Moco synthesis genes are affected by molybdate, and whether the endogenous levels of Moco vary, e.g., during plant development or in response to stress conditions. The only available information in this respect concerns the expression of molybdenum metabolism-related genes in the *Arabidopsis* *MOT1* mutant that was grown without additional molybdate supply to induce endogenous molybdate deficiency (Ide et al., 2011). In fact, the Moco biosynthesis genes *CNX2* and *CNX6* were demonstrated to be upregulated under molybdate deficiency, which was likewise true for the molybdo-enzymes *NR1* and *NR2*, the Moco sulfurase *ABA3* and the transporter *MOT2*. Apart from these genes, molybdate deficiency also affected the expression of many genes involved in transport, stress responses, signal tranduction and in the metabolisms of nitrogen, sulfur, and phosphate, but also the levels of amino acids, sugars, organic acids, and purine metabolites were significantly altered, indicating that molybdate nutrition has global impact on plants.

Among the molybdo-enzymes, nitrate reductase (NR) represents the cytosolic key enzyme of nitrogen assimilation that reduces nitrate to nitrite (Campbell, 2001). In addition to Moco, NR also depends on heme and FAD as prosthetic groups and strictly requires NADH or NADPH for enzymatic activity. A deficiency in NR results in the inability of the plant to mobilize nitrogen, which is inseparably associated with the loss of plant viability in the absence of alternative nitrogen sources. Under low-oxygen conditions, NR is capable of reducing nitrite to nitric oxide (NO), and NR-derived NO appears to be among the major sources of NO in plants with impact on plant development, protection against reactive oxygen species, phytoalexin accumulation, and pathogen resistance (Rockel et al., 2002).

Plant sulfite oxidase (SO) is a peroxisomal enzyme (Nowak et al., 2004), which exclusively consists of a Moco-binding domain required for oxidizing sulfite to sulfate. In this process, substrate-derived electrons are transferred to molecular oxygen with formation of superoxide anions (Byrne et al., 2009) and hydrogen peroxides (Haensch et al., 2006). In the physiological context, SO represents a part of an intracellular sulfite homeostasis network required to prevent plant cells from the toxic effects of endogenously arising sulfite (Brychkova et al., 2007; Lang et al., 2007).

Xanthine dehydrogenase (XDH) requires Moco, FAD, and two iron-sulfur clusters (Hille et al., 2011), and its main function is associated to purine degradation by oxidizing hypoxanthine to xanthine and xanthine to uric acid in the cytosol. Electrons released from the substrate are preferably transferred to NAD^+^. At extremely low concentrations of NAD^+^, molecular oxygen can serve as alternative electron acceptor with simultaneous generation of superoxides (Hesberg et al., 2004; Yesbergenova et al., 2005; Zarepour et al., 2010). As indicated by XDH-deficient plants, the function of XDH is crucial for plant growth, senescence, and fertility (Nakagawa et al., 2007; Brychkova et al., 2008). Independent from other substrates, XDH exhibits strong intrinsic NADH oxidase activity, which is accompanied by the use of oxygen as electron acceptor and simultaneous formation of superoxide anions (Yesbergenova et al., 2005; Zarepour et al., 2010). It is speculated that this activity is of importance in the response to biotic and abiotic stresses.

Aldehyde oxidase (AO) has derived from XDH by gene duplication and neo-functionalization (Rodríguez-Trelles et al., 2003) and therefore shares catalytical and structural similarities with XDH. In contrast to XDH however, AO proteins preferably oxidize aldehydes to the respective carboxylic acid. Moreover, molecular oxygen is the exclusive electron acceptor during catalysis and its consumption is obligatorily linked with the generation of hydrogen peroxide and superoxide anions (Yesbergenova et al., 2005; Zarepour et al., 2012). Obviously, the genome of most, if not all, plant species harbors several AO genes, which indicates a specific need of plants for several independent aldehyde oxidation activities. The number of AO proteins and their specific functions might therefore relate to the specific metabolic and environmental demands of different plant species. For instance, the *Arabidopsis* genome encodes the genes *AAO1* – *AAO4* and the respective proteins form homo- and hetero-dimers with overlapping, but also distinct substrate specificities (Akaba et al., 1999; Koiwai et al., 2000; Seo et al., 2000a,b). The most important isoform is AAO3, which catalyzes the oxidation of abscisic aldehyde to abscisic acid (ABA) in the last cytosolic step of ABA synthesis. Due to the function of ABA in many aspects of plant growth and development, and in adaptation to a variety of abiotic stresses, *AAO3*-deficient plants with reduced ABA levels are characterized by a high transpiration rate, reduced stress tolerance, and impaired seed dormancy (Seo and Koshiba, 2011). For AAO1 and AAO2, a function in one of the multiple biosynthesis routes of indole-3-acetic acid is suggested as they both efficiently catalyze the oxidation of indole acetaldehyde to indole-3-acetic acid *in vitro*. AAO4 is expressed preferably in siliques and catalyzes the oxidation of benzaldehyde into benzoic acid, the latter being incorporated into glucosinolates that likely serve as herbivore defense compounds (Ibdah et al., 2009).

The mitochondrial amidoxime reducing component (mARC) has been identified in mitochondria of mammals and catalyzes the reduction of *N*-hydroxylated substances (Havemeyer et al., 2011). Like mammals, plant genomes encode two mARC isoforms, which have not yet been investigated in detail. The physiological role of mARC proteins is therefore still enigmatic, even though previous studies in *Chlamydomonas *and on recombinant human proteins suggest a function in the detoxification of *N*-hydroxylated base analogs (Chamizo-Ampudia et al., 2011; Krompholz et al., 2012) and/or in the regulation of L-arginine-dependent NO synthesis (Kotthaus et al., 2011).

Although the physiological functions of some molybdo-enzymes are as yet not fully understood, it is obvious that others hold key positions in essential or at least important metabolic pathways. Any factor that affects one of these enzymes will thus also affect the respective pathway, with effects on Moco biosynthesis and molybdate supply resulting in the pleiotropic impairment of all molybdo-enzyme activities, associated with severe reduction of plant viability or even death of the plant.

## CROSSTALK BETWEEN MOLYBDENUM AND IRON METABOLISM

An interrelation between molybdenum and iron has been reported by Berry and Reisenauer (1967) who found that molybdate supply significantly increases the capacity of tomato plants to absorb Fe^2^^+^. The inverse phenomenon, an influence of Fe^2^^+^ on molybdate uptake, has later been investigated in excised rice roots, which showed increased molybdate uptake capacity in the presence of FeSO_4_ (Kannan and Ramani, 1978). This interrelation was confirmed in an ionomics study involving iron-deficient plants, in which molybdenum contents were shown to be reduced (Baxter, 2009). However, the link between the two metals in this approach is not fully understood and might be related either to acidification of the rhizosphere during iron-deficiency, which would result in molybdenum becoming less available to the plant, or to down-regulation of MOT1, which might affect molybdate uptake. Interestingly, several key genes involved in molybdenum metabolism were found to respond to iron-deficiency in *Arabidopsis* roots with the *MOT1* gene indeed showing down-regulation, and the genes *CNX2*, *CNX3, *and *ABA3 *showing**up-regulation (). By contrast, under molybdate-deficient conditions as induced by Ide et al. (2011) none of the known key genes of iron uptake and transport such as *FRO2*, *IRT1*, or *ferritins *were found to be altered in expression. Instead, only the genes of the ferric-chelate reductases oxidases FRO6 and FRO7 and the iron-regulated protein IREG1 showed down-regulation. It can thus be assumed that iron availability is a crucial regulatory element for plant molybdenum metabolism, while molybdate availability is of subordinated importance for iron metabolism.

Apart from metal uptake and gene regulation, crosstalk between molybdenum and iron is observed on the levels of Moco biosynthesis and molybdo-enyzmes. As previously mentioned, some of the latter require iron-containing prosthetic groups such as iron-sulfur clusters (AO and XDH) or heme (NR) besides Moco and FAD. Indeed, when looking in more detail on the more than 50 molybdo-enzymes known in all organisms, then the vast majority also depends on such iron-containing groups (Hille, 2013). It seems therefore reasonable to assume that this co-occurence of molybdenum and iron is due to the beneficial influence on each other’s redox properties. In addition, the Moco biosynthesis enzyme CNX2 requires two [4Fe-4S] clusters for activity and the crosstalk between molybdenum and iron is further substantiated by the fact that *CNX2* gene expression is controlled by both, molybdate and iron availability.

Molybdenum and iron metabolisms also merge at the function of ATM3, a mitochondrial ABC-type transporter that is crucial for the maturation of extra-mitochondrial iron-sulfur proteins (Bernard et al., 2009; Balk and Pilon, 2011). ATM3 exports an as yet unknown sulfur-containing compound from the mitochondria into the cytosol, where this compound is used for assembly of iron-sulfur clusters. Surprisingly, ATM3 has been found to play an important role in enabling the export of the Moco biosynthesis intermediate cPMP from mitochondria into the cytosol as ATM3-deficient plants present reduced levels of Moco accompanied by increased cPMP levels in mitochondria (Teschner et al., 2010). Since the two molecules requiring ATM3 for transport are likely to differ markedly, one can hypothesize that both are co-transported with glutathione, which has been speculated earlier to be a substrate of ATM3 (Kim et al., 2006) and to stimulate the activity of this type of transporter (Kuhnke et al., 2006).

Finally, an evolutionary overlap between molybdenum and iron metabolisms exists by the function of the Moco sulfurase ABA3, which is essentially required for the final activation of AO and XDH. ABA3 is a two-domain protein with a N-terminal L-cysteine desulfurase domain that decomposes L-cysteine to L-alanine and sulfur (Bittner et al., 2001; Heidenreich et al., 2005). The sulfur is bound as a persulfide to a strictly conserved cysteine residue of the protein (ABA3-Cys_430_SH + S^2^^-^ = > ABA3-Cys_430_S-SH) and transferred to the Moco-binding C-terminal domain (Wollers et al., 2008). On the C-terminal domain, the persulfide sulfur is transformed into a molybdenum-bound sulfido ligand by replacing an oxygen ligand [pterin-MoO_2_(OH) + S^2^^-^ = > pterin-MoOS(OH) + O^2^^-^]. With receiving this type of Moco, the target proteins of ABA3, AO, and XDH, finally gain activity. Interestingly, L-cysteine desulfurases originated from the more ancient iron-sulfur cluster biosynthesis, in which they abstract sulfur from L-cysteine and deliver it to various scaffold proteins, onto which iron-sulfur clusters are assembled prior to insertion into apo-enzymes (Lill et al., 2012). In case of ABA3 and its orthologs however, a cysteine desulfurase must have been fused to a selected scaffold to evolve into a new activator protein highly specific and exclusive for AO and XDH.

Recently, an interesting observation has been made concerning the effect of sulfur supply on AO and XDH proteins (Cao et al., 2013). In this work, sulfate deficiency has been found to impair endogenous cysteine levels and simultaneously, the biosynthesis of the phytohormone ABA. This effect has been ascribed to reduced activity of AAO3 and it was concluded that AAO3, and in addition also XDH, cannot properly be sulfurated by the Moco sulfurase ABA3 due to the limited availability of its substrate L-cysteine. This might indeed be one possible explanation for reduced activities of AO and XDH proteins in sulfate-deprived plants; yet, an effect on the biosynthesis of iron-sulfur clusters as required by AO and XDH proteins appears even more likely due to the higher demand of this process for L-cysteine (Forieri et al., 2013). As sulfur is embedded into molybdenum metabolism also at several other steps (e.g., Moco synthesis, sulfite detoxification, molybdate uptake), the three nutrients molybdenum, iron and sulfur appear to interact closely on various levels within a common metabolic network that needs to be elucidated in future studies.

## CONCLUSIONS AND PERSPECTIVES

Many players in the molybdenum homeostasis network have meanwhile been identified and characterized with the enzymes of Moco synthesis and the molybdo-enzymes representing the most intensively-studied topics, even though also here many aspects still remain to be solved. Understanding of the function of molybdate transporters has just begun and a lot more effort needs to be invested to depict the import and export routes in plants and to identify the candidate(s) responsible for uptake of molybdate at the root:soil interface. A point, which has not been considered before is whether molybdate-binding and/or -storage proteins exist in plants that might shuttle molybdate between organelles and tissues or store it for specific requirements, respectively. Moreover, very little is known about factors controlling the expression of genes and interaction of proteins involved in molybdenum homeostasis, thus requiring further in-depth analysis on the transcriptome, proteome, interactome, metabolome, and ionome level.

## Conflict of Interest Statement

The authors declare that the research was conducted in the absence of any commercial or financial relationships that could be construed as a potential conflict of interest.

## References

[B1] Akaba S. Seo M. Dohmae N. Takio K. Sekimoto H. Kamiya Y. (1999). Production of homo- and hetero-dimeric isozymes from two aldehyde oxidase genes of *Arabidopsis thaliana*. *J. Biochem.* 126 395–401 10423535 10.1093/oxfordjournals.jbchem.a022463

[B2] Balk J. Pilon M. (2011). Ancient and essential: the assembly of iron-sulfur clusters in plants. *Trends Plant Sci.* 16 218–226 10.1016/j.tplants.2010.12.006 21257336

[B3] Baxter I. (2009). Ionomics: studying the social network of mineral nutrients. *Curr. Opin. Plant Biol.* 12 381–386 10.1016/j.pbi.2009.05.002 19481970 PMC2701637

[B4] Baxter I. Muthukumar B. Park H. C. Buchner P. Lahner B. Danku J. (2008). Variation in molybdenum content across broadly distributed populations of *Arabidopsis thaliana* is controlled by a mitochondrial molybdenum transporter (MOT1). *PLoS Genet.* 4:e1000004 10.1371/journal.pgen.1000004 PMC2265440 18454190

[B5] Bernard D. G. Cheng Y. Zhao Y. Balk J. (2009). An allelic mutant series of ATM3 reveals its key role in the biogenesis of cytosolic iron-sulfur proteins in *Arabidopsis*. *Plant Physiol.* 151 590–602 10.1104/pp.109.143651 19710232 PMC2754654

[B6] Berry J. A. Reisenauer H. M. (1967). The influence of molybdenum on iron nutrition of tomato. *Plant Soil* 27 303–313

[B7] Bittner F. Mendel R. R. (2010). “Cell Biology of Molybdenum,” in *Cell Biology of Metals and Nutrients* eds. Hell R. Mendel R. R. (Springer-Verlag Berlin Heidelberg 2010), *Plant Cell Monogr.* 17 119–143 10.1007/978-3-642-10613-2_6

[B8] Bittner F. Oreb M. Mendel R. R. (2001). ABA3 is a molybdenum cofactor sulfurase required for activation of aldehyde oxidase and xanthine dehydrogenase in *Arabidopsis thaliana*. *J. Biol. Chem.* 276 40381–40384 10.1074/jbc.C100472200 11553608

[B9] Brychkova G. Alikulov Z. Fluhr R. Sagi M. (2008). A critical role for ureides in dark and senescence-induced purine remobilization is unmasked in the Atxdh1 *Arabidopsis* mutant. *Plant J.* 54 496–509 10.1111/j.1365-313X.2008.03440.x 18266920

[B10] Brychkova G. Xia Z. Yang G. Yesbergenova Z. Zhang Z. Davydov O. (2007). Sulfite oxidase protects plants against sulfur dioxide toxicity. *Plant J.* 50 696–709 10.1111/j.1365-313X.2007.03080.x 17425719

[B11] Byrne R. S. Haensch R. Mendel R. R. Hille R. (2009). Oxidative half-reaction of *Arabidopsis thaliana* sulfite oxidase: generation of superoxide by a peroxisomal enzyme. *J. Biol. Chem.* 284 35479–35484 10.1074/jbc.M109.067355 19875441 PMC2790977

[B12] Campbell W. H. (2001). Structure and function of eukaryotic NAD(P)H:nitrate reductase. *Cell Mol. Life Sci.* 58 194–204 10.1007/PL00000847 11289301 PMC11146515

[B13] Cao M. J. Wang Z. Zhao Q. Mao J. L. Speiser A. Wirtz M. (2013). Sulfate availability affects ABA levels and germination response to ABA and salt stress in *Arabidopsis thaliana*. *Plant J.* 10.1111/tpj.12407[Epub ahead of Print]. 24330104

[B14] Chamizo-Ampudia A. Galvan A. Fernandez E. Llamas A. (2011). The *Chlamydomonas reinhardtii* molybdenum cofactor enzyme crARC has a Zn-dependent activity and protein partners similar to those of its human homologue. *Eukaryot. Cell* 10 1270–1282 10.1128/EC.05096-11 21803866 PMC3187064

[B15] Forieri I. Wirtz M. Hell R. (2013). Toward new perspectives on the interaction of iron and sulfur metabolism in plants. *Front. Plant Sci.* 4:357 10.3389/fpls.2013.00357. PMC3788360 24106494

[B16] Gasber A. Klaumann S. Trentmann O. Trampczynska A. Clemens S. Schneider S. (2011). Identification of an *Arabidopsis* solute carrier critical for intracellular transport and inter-organ allocation of molybdate. *Plant Biol. (Stuttg).* 13 710–718 10.1111/j.1438-8677.2011.00448.x 21815974

[B17] Haensch R. Lang C. Riebeseel E. Lindigkeit R. Gessler A. Rennenberg H. (2006). Plant sulfite oxidase as novel producer of H2O2: combination of enzyme catalysis with a subsequent non-enzymatic reaction step. *J. Biol. Chem.* 281 6884–6888 10.1074/jbc.M513054200 16407262

[B18] Havemeyer A. Lang J. Clement B. (2011). The fourth mammalian molybdenum enzyme mARC: current state of research. *Drug Metab. Rev.* 43 524–539 10.3109/03602532.2011.608682 21942410

[B19] Heidenreich T. Wollers S. Mendel R. R. Bittner F. (2005). Characterization of the NifS-like domain of ABA3 from *Arabidopsis thaliana* provides insight into the mechanism of molybdenum cofactor sulfuration. *J. Biol. Chem.* 280 4213–4218 10.1074/jbc.M411195200 15561708

[B20] Hesberg C. Haensch R. Mendel R. R. Bittner F. (2004). Tandem orientation of duplicated xanthine dehydrogenase genes from *Arabidopsis thaliana*: differential gene expression and enzyme activities. *J. Biol. Chem.* 279 13547–13554 10.1074/jbc.M312929200 14726515

[B21] Hille R. (2013). The molybdenum oxotransferases and related enzymes. *Dalton Trans.* 42 3029–3042 10.1039/c2dt32376a 23318732

[B22] Hille R. Nishino T. Bittner F. (2011). Molybdenum enzymes in higher organisms. *Coord. Chem. Rev.* 255 1179–1205 10.1016/j.ccr.2010.11.034 21516203 PMC3079273

[B23] Hu Y. Ribbe M. W. (2013). Nitrogenase assembly. *Biochim. Biophys. Acta* 1827 1112–1122 10.1016/j.bbabio.2012.12.001 23232096 PMC3622157

[B24] Ibdah M. Chen Y.T. Wilkerson C.G. Pichersky E. (2009). An aldehyde oxidase in developing seeds of *Arabidopsis* converts benzaldehyde to benzoic acid. *Plant Physiol*. 150 416–423 10.1104/pp.109.135848 19297586 PMC2675751

[B25] Ide Y. Kusano M. Oikawa A. Fukushima A. Tomatsu H. Saito K. (2011). Effects of molybdenum deficiency and defects in molybdate transporter MOT1 on transcript accumulation and nitrogen/sulphur metabolism in *Arabidopsis thaliana*. *J. Exp. Bot.* 62 1483–1497 10.1093/jxb/erq345 21131548

[B26] Kaiser B. N. Gridley K. L. Ngaire Brady J. Phillips T. Tyerman S. D. (2005). The role of molybdenum in agricultural plant production. *Ann. Bot.* 96 745–754 10.1093/aob/mci226 16033776 PMC4247040

[B27] Kannan S. Ramani S. (1978). Studies on molybdenum absorption and transport in bean and rice. *Plant Physiol*. 62 179–181 10.1104/pp.62.2.179 16660481 PMC1092085

[B28] Kim D. Y. Bovet L. Kushnir S. Noh E. W. Martinoia E. Lee Y. (2006). AtATM3 is involved in heavy metal resistance in *Arabidopsis*. *Plant Physiol.* 140 922–932 10.1104/pp.105.074146 16461380 PMC1400565

[B29] Kneip C. Lockhart P. Voss C. Maier U. G. (2007). Nitrogen fixation in eukaryotes-new models for symbiosis. *BMC Evol. Biol.* 7:55 10.1186/1471-2148-7-55 PMC1853082 17408485

[B30] Koiwai H. Akaba S. Seo M. Komano T. Koshiba T. (2000). Functional expression of two *Arabidopsis* aldehyde oxidases in the yeast Pichia pastoris. *J. Biochem.* 127 659–664 10739959 10.1093/oxfordjournals.jbchem.a022654

[B31] Kotthaus J. Wahl B. Havemeyer A. Kotthaus J. Schade D. Garbe-Schoenberg D. (2011). Reduction of N(ω)-hydroxy-L-arginine by the mitochondrial amidoxime reducing component (mARC). *Biochem. J.* 433 383–391 10.1042/BJ20100960 21029045

[B32] Krompholz N. Krischkowski C. Reichmann D. Garbe-Schoenberg D. Mendel R. R. Bittner F. (2012). The mitochondrial amidoxime reducing component (mARC) is involved in detoxification of N-hydroxylated base analogues. *Chem. Res. Toxicol.* 25 2443–2450 10.1021/tx300298m 22924387

[B33] Kuhnke G. Neumann K. Muehlenhoff U. Lill R. (2006). Stimulation of the ATPase activity of the yeast mitochondrial ABC transporter Atm1p by thiol compounds. *Mol. Membr. Biol.* 23 173–184 16754360 10.1080/09687860500473630

[B34] Kumchai J. Huang J. Z. Lee C. Y. Chen F. C. Chin S. W. (2013). Proline partially overcomes excess molybdenum toxicity in cabbage seedlings grown in vitro. *Genet. Mol. Res.* 12 5589–5601 10.4238/2013.November.18.8 24301928

[B35] Lang C. Popko J. Wirtz M. Hell R. Herschbach C. Kreuzwieser J. (2007). Sulfite oxidase as key enzyme for protecting plants against sulfur dioxide. *Plant Cell Environ.* 30 447–455 10.1111/j.1365-3040.2006.01632.x 17324231

[B36] Lill R. Hoffmann B. Molik S. Pierik A. J. Rietzschel N. Stehling O. (2012). The role of mitochondria in cellular iron-sulfur protein biogenesis and iron metabolism. *Biochim. Biophys. Acta* 1823 1491–1508 10.1016/j.bbamcr.2012.05.009 22609301

[B37] Mendel R. R. (2013). The molybdenum cofactor. *J. Biol. Chem.* 288 13165–13172 10.1074/jbc.R113.455311 23539623 PMC3650355

[B38] Nakagawa A. Sakamoto S. Takahashi M. Morikawa H. Sakamoto A. (2007). The RNAi-mediated silencing of xanthine dehydrogenase impairs growth and fertility and accelerates leaf senescence in transgenic *Arabidopsis* plants. *Plant Cell Physiol.* 48 1484–1495 10.1093/pcp/pcm119 17872919

[B39] Nowak K. Luniak N. Witt C. Wuestefeld Y. Wachter A. Mendel R. R. (2004). Peroxisomal localization of sulfite oxidase separates it from chloroplast-based sulfur assimilation. *Plant Cell Physiol.* 45 1889–1894 10.1093/pcp/pch212 15653809

[B40] Rockel P. Strube F. Rockel A. Wildt J. Kaiser W. M. (2002). Regulation of nitric oxide (NO) production by plant nitrate reductase in vivo and in vitro. *J. Exp. Bot.* 53 103–110 10.1093/jexbot/53.366.103 11741046

[B41] Rodríguez-Trelles F. Tarrío R. Ayala F. J. (2003). Convergent neofunctionalization by positive darwinian selection after ancient recurrent duplications of the xanthine dehydrogenase gene. *Proc. Natl. Acad. Sci. U.S.A.* 100 13413–13417 10.1073/pnas.1835646100 14576276 PMC263828

[B42] Seo M. Koiwai H. Akaba S. Komano T. Oritani T. Kamiya Y. (2000a). Abscisic aldehyde oxidase in leaves of *Arabidopsis thaliana*. *Plant J.* 23 481–488 10.1046/j.1365-313x.2000.00812.x 10972874

[B43] Seo M. Peeters A. J. Koiwai H. Oritani T. Marion-Poll A. Zeevaart J. A. (2000b). The *Arabidopsis* aldehyde oxidase 3 (AAO3) gene product catalyzes the final step in abscisic acid biosynthesis in leaves. *Proc. Natl. Acad. Sci. U.S.A.* 97 12908–12913 10.1073/pnas.220426197 11050171 PMC18863

[B44] Seo M. Koshiba T. (2011). Transport of ABA from the site of biosynthesis to the site of action. *J. Plant Res.* 124 501–507 10.1007/s10265-011-0411-4 21416315

[B45] Shibagaki N. Grossman A. R. (2006). The role of the STAS domain in the function and biogenesis of a sulfate transporter as probed by random mutagenesis. *J. Biol. Chem.* 281 22964–22973 10.1074/jbc.M603462200 16754669

[B46] Shinmachi F. Buchner P. Stroud J. L. Parmar S. Zhao F. J. McGrath S. P. (2010). Influence of sulfur deficiency on the expression of specific sulfate transporters and the distribution of sulfur, selenium, and molybdenum in wheat. *Plant Physiol.* 153 327–336 10.1104/pp.110.153759 20219830 PMC2862427

[B47] Stout P. R. Meagher W. R. Pearson G. A. Johnson C. M. (1951). Molybdenum nutrition of crop plants. I. The influence of phosphate and sulfate on the absorption of molybdenum from soils and solution cultures. *Plant Soil* 3 51–87

[B48] Tejada-Jiménez M. Galván A. Fernández E. (2011). Algae and humans share a molybdate transporter. *Proc. Natl. Acad. Sci. U.S.A.* 108 6420–6425 10.1073/pnas.1100700108 21464289 PMC3080982

[B49] Tejada-Jimenez M. Llamas A. Sanz-Luque E. Galvan A. Fernandez E. (2007). A high-affinity molybdate transporter in eukaryotes. *Proc. Natl. Acad. Sci. U.S.A.* 104 20126–20130 10.1073/pnas.0704646104 18077439 PMC2148433

[B50] Teschner J. Lachmann N. Geisler M. Selbach K. Balk J. Mendel R. R. (2010). A novel role for mitochondrial ABC transporter ATM3 from *Arabidopsis* in molybdenum cofactor biosynthesis. *Plant Cell* 22 468–480 10.1105/tpc.109.068478 20164445 PMC2845412

[B51] Tomatsu H. Takano J. Takahashi H. Watanabe-Takahashi A. Shibagaki N. Fujiwara T. (2007). An *Arabidopsis thaliana* high-affinity molybdate transporter required for efficient uptake of molybdate from soil. *Proc. Natl. Acad. Sci. U.S.A.* 104 18807–18812 10.1073/pnas.070637310 18003916 PMC2141858

[B52] Wollers S. Heidenreich T. Zarepour M. Zachmann D. Kraft C. Zhao Y. (2008). Binding of sulfurated molybdenum cofactor to the C-terminal domain of ABA3 from *Arabidopsis thaliana* provides insight into the mechanism of molybdenum cofactor sulfuration. *J. Biol. Chem.* 283 9642–9650 10.1074/jbc.M708549200 18258600

[B53] Yesbergenova Z. Yang G. Oron E. Soffer D. Fluhr R. Sagi M. (2005). The plant Mo-hydroxylases aldehyde oxidase and xanthine dehydrogenase have distinct reactive oxygen species signatures and are induced by drought and abscisic acid. *Plant J.* 42 862–876 10.1111/j.1365-313X.2005.02422.x 15941399

[B54] Zarepour M. Kaspari K. Stagge S. Rethmeier R. Mendel R. R. Bittner F. (2010). Xanthine dehydrogenase AtXDH1 from *Arabidopsis thaliana* is a potent producer of superoxide anions via its NADH oxidase activity. *Plant Mol. Biol.* 72 301–310 10.1007/s11103-009-9570-2 19915948

[B55] Zarepour M. Simon K. Wilch M. Nielaender U. Koshiba T. Seo M. (2012). Identification of superoxide production by *Arabidopsis thaliana* aldehyde oxidases AAO1 and AAO3. *Plant Mol. Biol.* 80 659–671 10.1007/s11103-012-9975-1 23065119

